# Community-based surveillance in internally displaced people’s camps and urban settings during a complex emergency in Yemen in 2020

**DOI:** 10.1186/s13031-021-00394-1

**Published:** 2021-07-05

**Authors:** Manal Salem Omar Baaees, Jeremias D. Naiene, Ali Ahmed Al-Waleedi, Nasreen Salem Bin-Azoon, Muhammad Fawad Khan, Nuha Mahmoud, Altaf Musani

**Affiliations:** 1 World Health Organization, Sana’a, Yemen; 2 Ministry of Public Health and Population, Aden, Yemen

**Keywords:** Community-based surveillance, IDP camps, Urban settings, COVID-19, Complex emergency, Yemen

## Abstract

**Background:**

The need for early identification of coronavirus disease (COVID-19) cases in communities was high in Yemen during the first wave of the COVID-19 epidemic because most cases presenting to health facilities were severe. Early detection of cases would allow early interventions to interrupt the transmission chains. This study aimed to describe the implementation of community-based surveillance (CBS) in in internally displaced people (IDP) camps and urban settings in Yemen from 15 April 2020 to 30 September 2020.

**Methods:**

Following the Centers for Disease Control and Prevention guidance for evaluation of surveillance systems, we assessed the usefulness and acceptability of CBS. For acceptability, we calculated the proportion of trained volunteers who reported disease alerts. To assess the usefulness, we compared the alerts reported through the electronic diseases early warning system (eDEWS) with the alerts reported through CBS and described the response activities implemented.

**Results:**

In Al-Mukalla City, 18% (14/78) of the volunteers reported at least one alert. In IDP camps, 58% (18/31) of volunteers reported at least one alert. In Al-Mukalla City, CBS detected 49 alerts of influenza-like illness, whereas health facilities detected 561 cases of COVID-19. In IDP camps, CBS detected 91 alerts of influenza-like illness, compared to 10 alerts detected through eDEWS. In IDP camps, CBS detected three other syndromes besides influenza-like illness (febrile illness outbreak suspicion, acute diarrhoea, and skin disease). In IDP camps, public health actions were implemented for each disease detected and no further cases were reported.

**Conclusions:**

In Yemen, CBS was useful for detecting suspected outbreaks in IDP camps. CBS implementation did not yield expected results in general communities in urban areas in the early stage of the COVID-19 pandemic when little was known about the disease. In the urban setting, the system failed to detect suspected COVID-19 cases and other diseases despite the ongoing outbreaks reported through eDEWS. In Yemen, as in other countries, feasibility and acceptability studies should be conducted few months before CBS expansion in urban communities. The project should be expanded in IDP camps, by creating COVID-19 and other disease outbreak reporting sites.

**Supplementary Information:**

The online version contains supplementary material available at 10.1186/s13031-021-00394-1.

## Background

When the novel coronavirus disease (COVID-19) epidemic was confirmed in Yemen on 10 April 2020, the country had an insufficient number of trained rapid response teams (RRTs) to investigate the suspected cases because of concurrent disease outbreaks, including cholera, diphtheria, arboviral diseases and others [1, 2]. One RRT was available in each district to cover more than 300,000 people, and most of the COVID-19 cases did not report to the health facilities [1, 3].

The surveillance system in Yemen is exclusively based on the electronic diseases early warning system (eDEWS), in which most health facilities are used as reporting sites for epidemic-prone diseases. Data from the health facilities are reported in real-time and visualized at all levels, allowing early detection of abnormal disease trends [3]. The rumours from the communities are detected by the governorate health office (GHO) through information provided by general public, media, and others. After rumour detection, the GHO deploys RRTs for investigation and response [1, 4]. RRTs are Ministry of Public Health and Population (MOPHP) staff supported by partners, each team comprising a physician, surveillance officer, laboratory technician, environmental health officer and a risk communication officer [1]. The initial response of the RRTs may include health promotion, chlorination of water, search and removal of mosquito breeding sites, and case management of mild cases, depending on the disease investigated [4]. The rumours investigated by RRTs are also reported through eDEWS and published in the weekly bulletins produced by the MOPHP.

More than 40,000 community volunteers recruited by national and international organizations were available in Yemen, and they mainly implemented health promotion activities [4]. These volunteers were also encouraged to report rumours of suspected outbreaks to the GHO to deploy RRTs [3].

Active community surveillance is more effective in identifying missed cases during a disease outbreak than passive surveillance, which is more useful before an outbreak [5–8]. Active community surveillance to detect missed epidemic-prone disease cases can be useful in Yemen, where 42% of the population require at least a 1-h drive to reach a hospital [9]. Event-based surveillance, including community-based surveillance (CBS) may allow the earliest detection of outbreaks, especially cases not reported to the health facilities [10].

Cases among internally displaced people (IDP) may easily be missed owing to limited access to health facilities for self-reporting [11, 12]. Because of the ongoing armed conflict and natural disasters in Yemen, around 172,386 IDPs were present in different governorates in the country from January to December 2020 [13].

Several approaches of CBS have been used in developing countries [6, 14, 15]. However, there is no specific standard approach for CBS implementation, although more than 79 unique surveillance systems have been described in developing countries over the years [16]. To increase the chances of detecting COVID-19 cases in communities in Yemen, an active CBS was performed by the community volunteers from MOPHP and partner organizations in urban communities, and in IDP camps. The active CBS was done through door-to-door visits targeting mainly people with influenza-like illness, unexplained cluster of diseases, and acute public health events. The general community health volunteers from urban area (GCHVs) and IDP community health volunteers (ICHVs) reported to their health facility focal points (HFFPs) the cases detected through active CBS. The HFFPs requested the RRTs deployment by the GHO when the cases met COVID-19 case definition, or cluster of cases or deaths. The cases not fulfilling the above-mentioned criteria were referred to the nearest health facilities for treatment without deployment of RRTs. Suspected COVID-19 cases in Yemen were defined as any person with influenza-like illness and history of travel or residence in affected areas in the past 14 days before the symptom onset or any case of severe influenza-like illness without a precise diagnosis. Confirmed cases were cases with polymerase chain reaction test positive for COVID-19 infection [1]. Herein, we describe the implementation of CBS in IDP camps and urban settings in Yemen from 15 April 2020 to 30 September 2020. To our knowledge, this is the first documentation of CBS implementation in Yemen, and it provided evidence at the national level. The challenges and lessons learned can be considered before further expanding the project in Yemen and other countries with similar contexts.

## Methods

### Setting

**Fig. 1:**
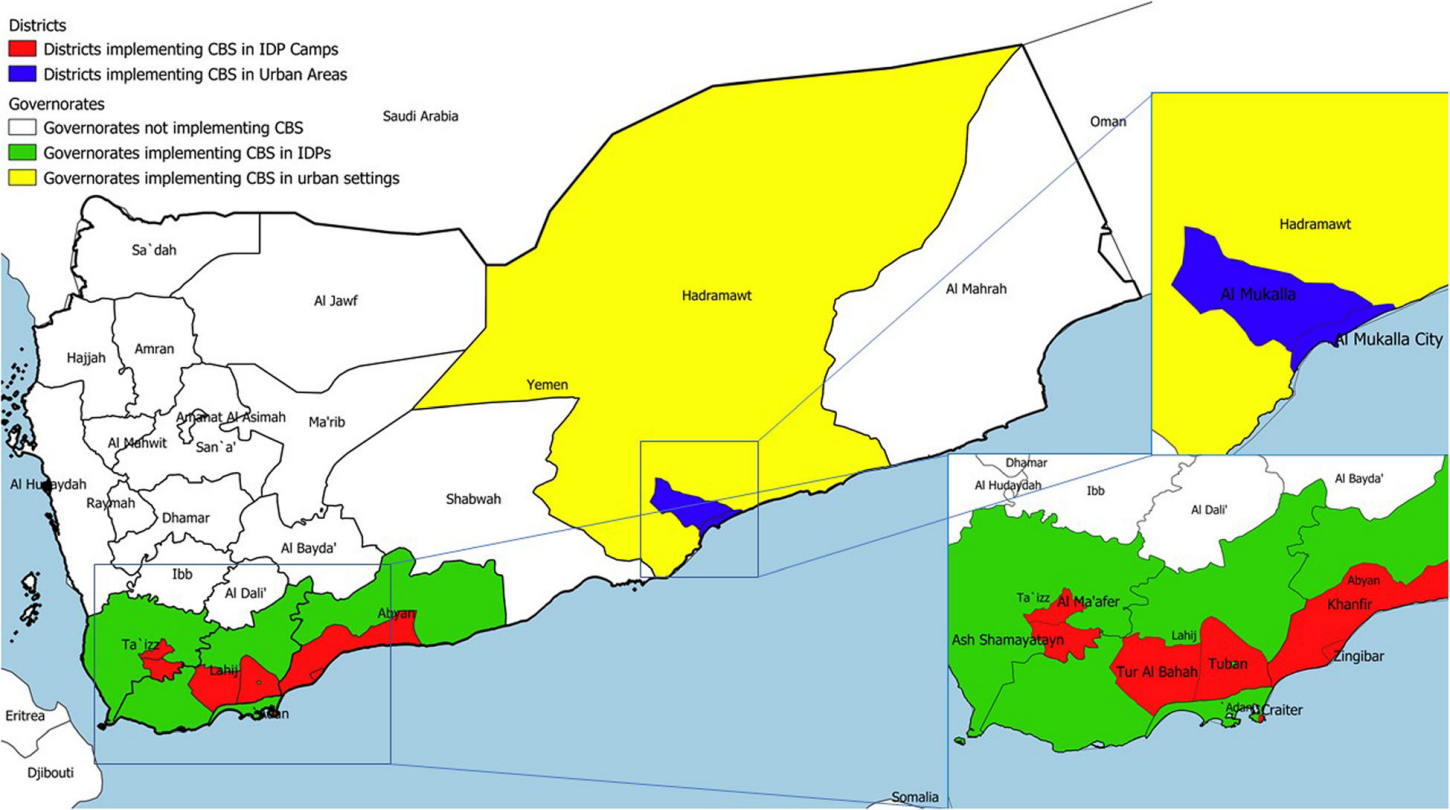
Areas of implementation of community-based surveillance pilot in Yemen, 2020

The Republic of Yemen is a country in the Arabian Peninsula that is subdivided into 23 governorates. The governorates in Yemen are further subdivided into 333 districts [17]. Among the governorates, CBS in IDP camps was piloted in Aden, Abyan, Lahj, and Taiz, while Hadramout piloted CBS in urban setting (Fig. 1) [17–19]. This paper describes the CBS pilot in Yemen in all the governorates and districts where it was implemented.

**Fig. 2:**
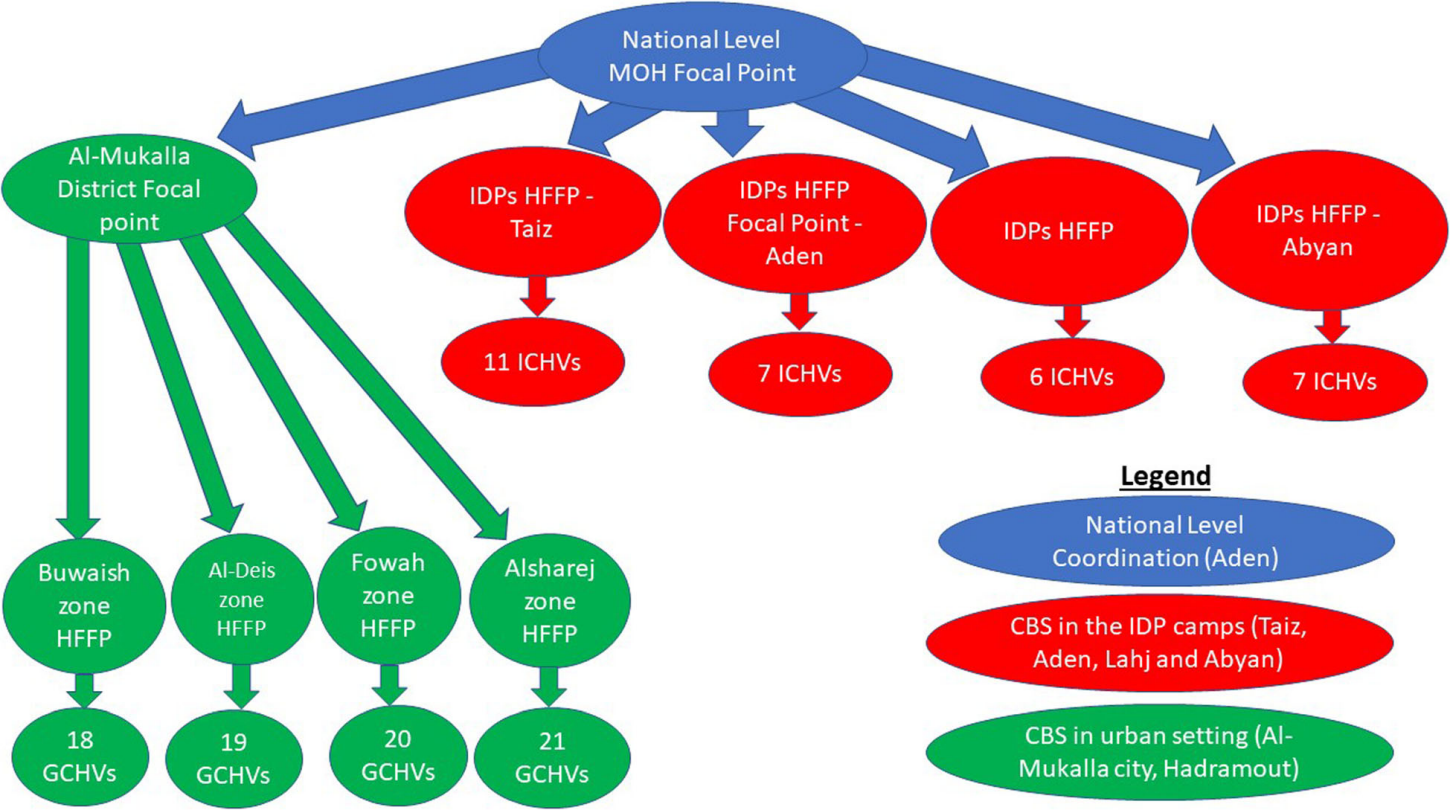
Final structure of community-based surveillance pilot, Yemen, 2020

Al-Mukalla city district in Hadramout governorate was selected for CBS piloting to target the general population in urban settings as part of a public health response to the first case of COVID-19 in Yemen. Although the case was reported in As Shikhr district, most of the patient’s contacts were in Al-Mukalla city. Among the 10 zones, we performed CBS in four zones wherein the contacts of the index cases were located (Fig. 2).

Al-Mukalla city is an urban area and one of the 28 districts of Hadramout governorate, which has 10 zones [18]. In 2017, the total population in Al-Mukalla city was estimated to be 264,100 inhabitants projected from the 2004 census, with an average of 7 people per household [18, 20]. The four zones selected for CBS pilot had a total population of 97,175 inhabitants (Table 1).

**Table 1:** Trained volunteers in CBS in Hadramout, Yemen, 2020

Zone	Population	Number of volunteers
Buwaish	6177	18
Fowa	13,168	20
Al-Deis	33,226	19
Alsharej	44,604	21
Total	97,175	78

*CBS* community-based surveillance

The CBS pilot targeting the IDP camps was performed in Aden, Lahj, Abyan, and Taizz because these governorates were accessible. Among the six governorates with the highest number of IDPs in Yemen, Marib, Al-Dhale’e, and Hudaydah were not easily accessible from Aden owing to ongoing armed conflicts. In each of the selected governorates, we selected the two districts with higher numbers of IDP camps and households, except Aden where IDP camps were concentrated in only one district, resulting in a total of seven districts selected for CBS (Table 2).

**Table 2:** Number of staff trained and CBS implementation in IDP camps by district in Yemen, 2020

District	Population in IDP camps	Number of IDP camps	Number of IDP households^a^	Number of IDP households in Camps^b^	Average number of households per camp	Number of volunteers trained^c^	Number of ICHVs trained	Number of volunteers who submitted at least one report^d^	Status of CBS^e^
	**Taizz Governorate**								
Ash-Shamayatayn	182	7	45	26	4	18	6	2	Activated
Al Ma’afer	119	6	66	17	3	6	5	3	Activated
Other Districts	490	12	1505	70	6	42	12	0	Not Activated
	**Abyan Governorate**								
Zingibar	210	2	139	30	15	2	2	1	Activated
Khanfir	406	5	86	58	12	5	5	2	Activated
Other Districts	119	4	702	17	4	11	11	0	Not Activated
	**Lahj Governorate**								
Tuban	84	5	23	12	2	5	4	3	Activated
Al Hawtah	105	2	15	15	8	3	2	2	Activated
Other Districts	140	0	795	20	0	11	0	0	Not Activated
	**Aden Governorate**								
Craiter	700	5	114	100	20	12	7	5	Activated
Other Districts	28	2	131	4	2	15	5	0	Not Activated

*IDP* internally displaced persons, *CBS* community-based surveillance, *ICHV* IDP camp community health volunteer

^a^Includes households outside of IDP camps, mixed with general communities, such as in private houses, host family houses, or second homes. Each household had an average of 7 people

^b^includes tents, public, and private buildings

^c^The training also targeted volunteers to cover IDPs in the host communities and refugees, although they did not submit any report and CBS was not activated in those areas

^d^Only ICHVs submitted reports, and not reports were submitted by the volunteers that were supposed to cover the IDPs in the host communities and refugees

^e^Despite the trainings targeting all the districts from the 4 governorates, only the 2 districts with a higher number of households were selected to activate CBS

According to the International Organization for Migration (IOM)‘s rapid displacement tracking (RDT) system, Marib, Al-Dhale’e, Taizz, Hudaydah, Lahj, and Abyan were the six governorates in Yemen with higher numbers of IDPs owing to active ongoing armed conflict. In the beginning of CBS project, on 15 April 2020, 71,304 IDP (11,884 households) were present in Yemen, and 14% of them were accommodated in tents, schools, and public buildings [13]. The others were welcomed by host families or rent houses in the general community. The seven districts that activated CBS had 32 IDP camps with a total population of 1806 inhabitants residing in the camps (Table 2).

### Alert triggers

To make the CBS as simple as possible, while keeping it sensitive enough to detect suspected COVID-19 cases without missing other health-related problems in the community, the GCHVs and ICHVs were requested to report only two types of alerts. These include alerts of influenza-like illness and unexplained clusters of diseases or public health events. The standard case definitions were simplified into community case definition used as an alert trigger. The influenza-like illnesses community case definition was “any case of acute onset of fever, cough, and difficult breathing”. The community case definition for unexplained clusters of diseases or public health events was defined as “unknown community health problems affecting two or more people”.

### Data source and analysis

We examined the lists of focal points and volunteers provided by MOPHP and partners. We matched the abovementioned lists with attendance lists of the trainings to identify the number of people trained for CBS. Moreover, we examined all alert triggers, line lists, weekly narrative, weekly aggregated and training reports produced from 15 April 2020 to 30 September 2020. This report describes the selection process, including the number of volunteers selected, selection criteria, and distribution of households per volunteer. We also describe the training timeliness, topics covered, the number of people trained, active CBS and referral process, incentives and personal protective equipment (PPEs) provided, and reporting system. Summary statistics, including the number of cases, distribution by sex, age group, geographical location, epidemiological week, and proportion of severe cases, were calculated. Data in the line lists were compiled and analysed using Microsoft Excel. We manually extracted data of confirmed COVID-19 cases in Yemen from the daily reports available from the official twitter account of the MOPHP. Weekly narrative reports were produced by the HFFPs and highlighted in bullet points to their challenges and the volunteers under their supervisions. We also examined the Yemen eDEWS bulletins from week 19, 2020 to week 40, 2020 to extract the number of alerts detected through the system. The bulletins were publicly available on the Yemen Health Cluster website. The IDP demographic information was obtained from the dataset of the RDT system from 1 January 2020 to 4 July 2020, which was available publicly in the IOM website. From this dataset, we extracted the data of IDPs that were present when the first alert trigger was submitted by the ICHVs on 28 June 2020.

### Attributes of CBS system

We followed the steps suggested in the World Health Organization (WHO) regional office for Africa guide for establishing a CBS in Yemen due to unavailability of specific guidelines for developing countries from the Eastern Mediterranean region [21]. We used the Centers for Disease Control and Prevention guidance for evaluation of surveillance systems to select the attributes of CBS presented in our report [22]. Based on the information collected during the CBS implementation in Yemen, we selected the attributes acceptability and usefulness.

#### Acceptability

We considered the acceptability of CBS as the willingness of the volunteers, health facility focal points, local authorities, organizations and the public to participate in the project [22]. To assess the acceptability, we calculated the proportion of ICHVs and GCHVs who submitted at least one alert trigger to the HFFPs. We also calculated the reporting rate of weekly aggregated reports, and weekly narrative reports submitted by the HFFPs to the governorate level. To estimate the number of weekly aggregated reports and weekly narrative reports that were expected to be submitted by the HFFPs, we multiplied the number of HFFPs by the number of weeks of CBS implementation. Therefore, the four HFFPs from IDP camps were expected to submit 52 weekly reports from the time of CBS activation in IDP camps on week 27, 2020 to the end of the project on week 40, 2020. The four HFFPs from Al-Mukalla City were expected to submit 84 weekly reports from the activation of CBS in an urban setting in week 19, 2020 to week 40, 2020.

#### Usefulness

We considered CBS useful if it satisfactorily detected suspected outbreaks and if the data collected led to the implementation of public health actions to control outbreaks [22]. To assess the ability of the CBS to detect outbreaks, we compared the number of alerts reported to the eDEWS with the alerts reported by ICHVs and GCHVs in the districts where CBS was activated. We also described the public health actions taken for cases detected by ICHVs and GCHVs to assess the usefulness of CBS. To understand the impact of public health actions, we counted the number of cases reported by the ICHVs and GCHVs after the actions were taken.

## Results

### Key interventions

#### Selection of CBS focal points and volunteers

Community volunteers previously conducting community for development (C4D) activities under the United Nations Children’s Fund (UNICEF) were selected to perform CBS activities in urban setting. Community volunteers previously conducting health promotion activities under the Camp Coordination and Camp Management (CCCM) cluster partners were selected to perform CBS activities in IDP camps. The volunteers were previously conducting longstanding activities and they were selected to perform CBS activities in their area of residence. A total of 80 community volunteers supported by UNICEF were available and initially selected by the GHO from Hadramout to cover the four zones in Al-Mukalla City, namely Al Share, Al-Deis, Buwaish and Fowah zones, with 20 volunteers in each zone. According to the number of houses in the zones, each volunteer would cover 180 houses, each of them to be visited monthly. A total of 130 ICHVs were identified and selected by the CCCM cluster partners in coordination with the Executive Unit of IDPs, the governmental unit responsible for linking the government with the organizations supporting IDPs. This allowed each IDP camp to be covered by at least one ICHV, regardless of the number of households in the camp. Therefore, the number of households per ICHV ranged from 2 to 20 (Table 2). Any person able to read and write, aged more than 18 years, residing in the community or IDP camp and previously performing other health related tasks such as C4D or health promotion activities, was eligible to be GCHV or ICHV. The HFFPs from Al-Mukalla city were selected by the GHO from Hadramout, while the MOPHP national CBS coordinator selected the HFFPs from the IDP camps. Any person with epidemiological background was eligible for HFFP.

#### Training of governorate, district, health facility and community level staff

**Fig. 3:**
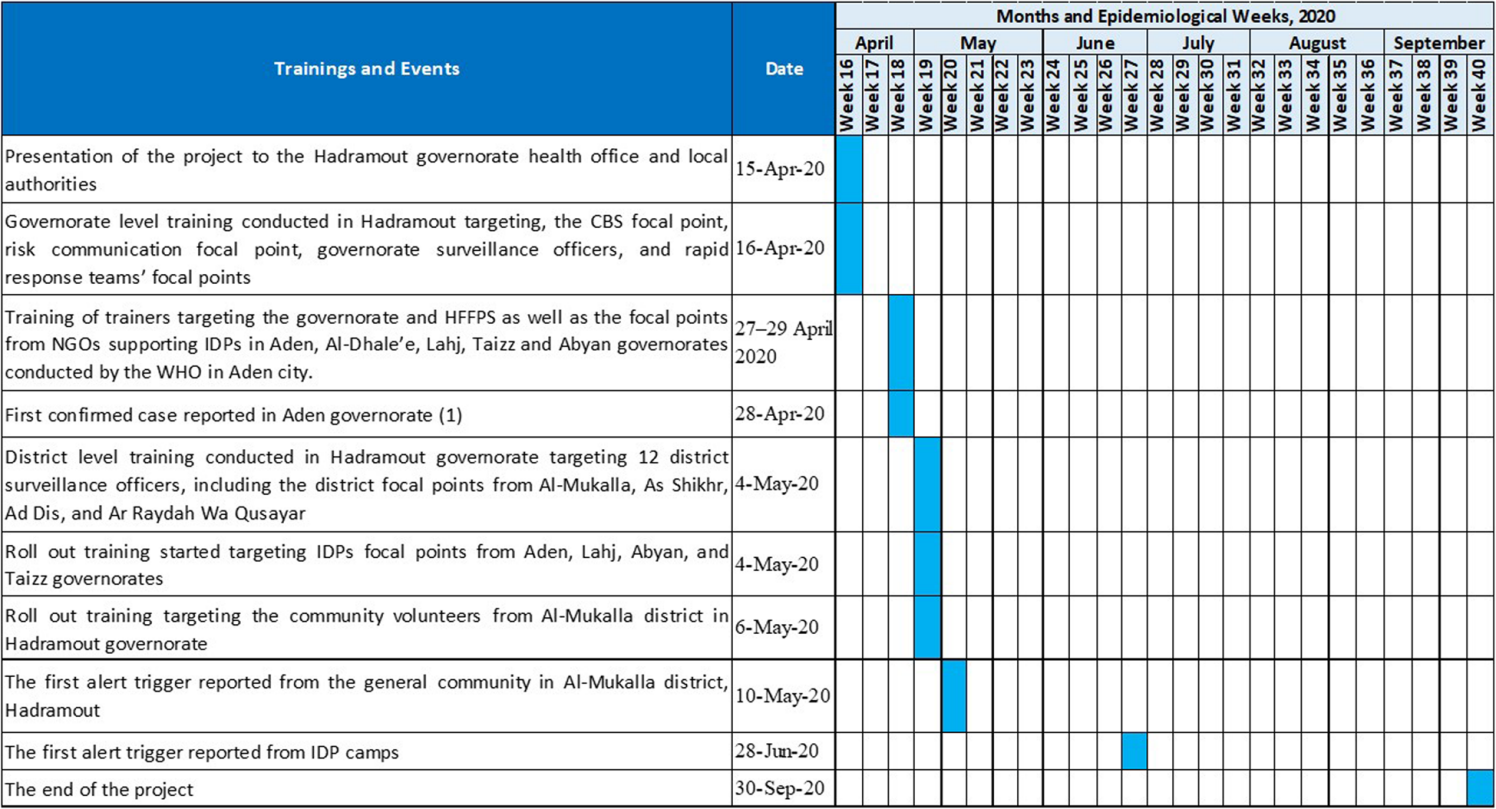
Chronology of the main trainings and events related to CBS in Yemen, 2020

On 16 April 2020, the WHO conducted a one-day training of CBS in urban setting governorate level team trainers. The roll out training was conducted by the governorate focal point on 4 May 2020 and targeted district surveillance officers and HFFPs from Al-Mukalla city and three other districts in Hadramout governorate. From 27 to 29 April 2020, IDP camp HFFPs from Aden, Abyan, Lahj, and Taiz were trained. The ICHVs were trained from 4 to 6 May 2020 by HFFPs, and the GCHVs were trained from 6 to 8 May 2020 (Fig. 3). The training for both CBS in urban settings and IDP camps covered the basics of CBS, including key definitions, objectives, structure, roles and responsibilities, strategy, and others. The training also covered overview of respiratory illnesses, community case definitions, active CBS, reporting tools, and timeliness of notification and reporting. Brochures with health education messages and alert triggers were printed and distributed to all GCHVs, ICHVs and focal points.

A total of 14 district level staff from four districts were trained in CBS in Hadramout governorate. Additionally, 78 of 80 GCHVs selected in Al-Mukalla city district were trained, of whom 67% (*n* = 52) were women (Tables 3 and 4). Moreover, 175 ICHVs were trained, of whom 31 were assigned to be ICHVs in areas where CBS was activated. Of the ICHVs trained, 74% (*n* = 130) were men. All HFFPs selected for community surveillance in IDP camps were medical doctors, whereas those in Hadramout governorate were non-clinicians with basic training in public health surveillance.

**Table 3:** Distribution of the staff trained in CBS by district in Hadramout, Yemen, 2020

	District				
	Al-Mukalla city	As Shikhr	Ad Dis	Ar Raydah Wa Qusayar	Total
District surveillance officers trained in CBS	1	1	1	1	**4**
Health facility focal points trained	4	2	2	2	**10**
Community volunteers trained on CBS^a^	78	0	0	0	**78**
Number of zones selected for CBS	4	0	0	0	**4**
Hadramout governorate level surveillance officers trained in CBS	NA	NA	NA	NA	**6**^b^
Status of CBS	Activated	Not Activated	Not Activated	Not Activated	

*CBS* community-based surveillance, *NA* not applicable

^a^20 volunteers trained for each zone, except two zones were 19 volunteers were trained in each

^b^Only includes the surveillance officers trained at the governorate level, excluding the ones trained at the district level

**Table 4:** Demographic characteristics of staff trained in CBS in Yemen, 2020

Governorate	District	Zone/Site	Level	Function	Gender			Background			
					Male	Female	Total	Medical Doctor	Nurse	Pharmacist	Others^a^
Aden	NA	NA	National	CBS Coordinator	0	1	1	1	0	0	0
**General CBS**											
Hadramout	Al-Mukalla	NA	Governorate^b^	CBS Coordinator	1	0	1	0	0	0	1
Hadramout	Al-Mukalla	Buwaish	Zone	HFFP	1	0	1	0	1	0	0
Hadramout	Al-Mukalla	Al-Deis	Zone	HFFP	1	0	1	0	0	1	0
Hadramout	Al-Mukalla	Fowa	Zone	HFFP	0	1	1	0	1	0	1
Hadramout	Al-Mukalla	Alsharej	Zone	HFFP	0	1	1	0	0	0	1
Hadramout	Al-Mukalla	Buwaish	Community	GCHV	7	11	18	0	0	0	18
Hadramout	Al-Mukalla	Al-Deis	Community	GCHV	10	9	19	0	0	0	19
Hadramout	Al-Mukalla	Fowa	Community	GCHV	7	13	20	0	0	0	20
Hadramout	Al-Mukalla	Alsharej	Community	GCHV	2	19	21	0	0	0	21
**CBS in IDPs/Refugee sites**											
Aden	NA	NA	Governorate	HFFP	1	0	0	1	0	0	0
Lahj	NA	NA	Governorate	HFFP	1	0	0	1	0	0	0
Abyan	NA	NA	Governorate	HFFP	1	0	0	1	0	0	0
Taizz	NA	NA	Governorate	HFFP	1	0	0	1	0	0	0
Abyan	Zingibar and Khanfir	NA	IDP camp	ICHV	14	4	18	0	0	0	18
Aden	Craiter	NA	IDP camp	ICHV	18	9	27	0	0	0	27
Lahj	Tuban and Al Hawtah	NA	IDP camp	ICHV	11	8	19	0	0	0	19
Al-Dhale’e	Ad-Dhale, Qa’atabah, Al-Hussein, Al-Azarik	NA	Community	NA^c^	26	3	29	0	0	0	29
Hudaydah	Khokha, Tuhayta	NA	Community	NA^c^	13	3	16	0	0	0	16
Taizz	Ash-Shamayatayn and Almaafer	NA	IDP camp	ICHV	48	18	66	0	0	0	66

*NA* not applicable, *CBS* community-based surveillance, *IDP* internally displaced person, *GCHV* general community health volunteer, *HFFP* health facility focal point, *ICHV* IDP camp community health volunteer

^a^Paramedics, physician assistants, and non-clinicians (including a bachelor’s degree in public health) with a formal training in public health surveillance

^b^Was in charge also for Al-Mukalla district because it was the only district activating CBS in the Hadramout governorate

^c^The volunteers were trained to work in CBS targeting the IDP in host communities outside of the camps, but CBS was not activated in this target groups

### Organisation of CBS and referral

GCHVs and ICHVs actively searched for suspected COVID-19 cases and unusual cluster of diseases in communities and IDP camps respectively through daily door-to-door visits and weekly community meetings. The GCHVs and ICHVs were requested to conduct weekly meetings with the communities to auscultate the main challenges faced by the communities and to detect cases missed during door-to-door visits. Moreover, the GCHVs and ICHVs performed health education activities during the door-to-door visits and community meetings. Transportation allowance was provided by the WHO to all trained GCHVs and ICHVs to facilitate their movement. The allowance for GCHVs and ICHVs was calculated based on the rate applied by MOPHP and the distance travelled. UNICEF and CCCM partners provided PPEs to the GCHVs and ICHVs respectively for the activities that they had previously performed before the CBS pilot, including C4D and health promotion activities. These PPEs included masks, gloves, face shields, and gowns. Further, they were also used for CBS activities as no additional PPE was available due to the global shortage. The GCHVs and ICHVs were oriented to follow all preventive measures, including physical distancing, always wearing PPEs while interacting with household members and not entering the houses.

### Data transmission and feedback

**Fig. 4:**
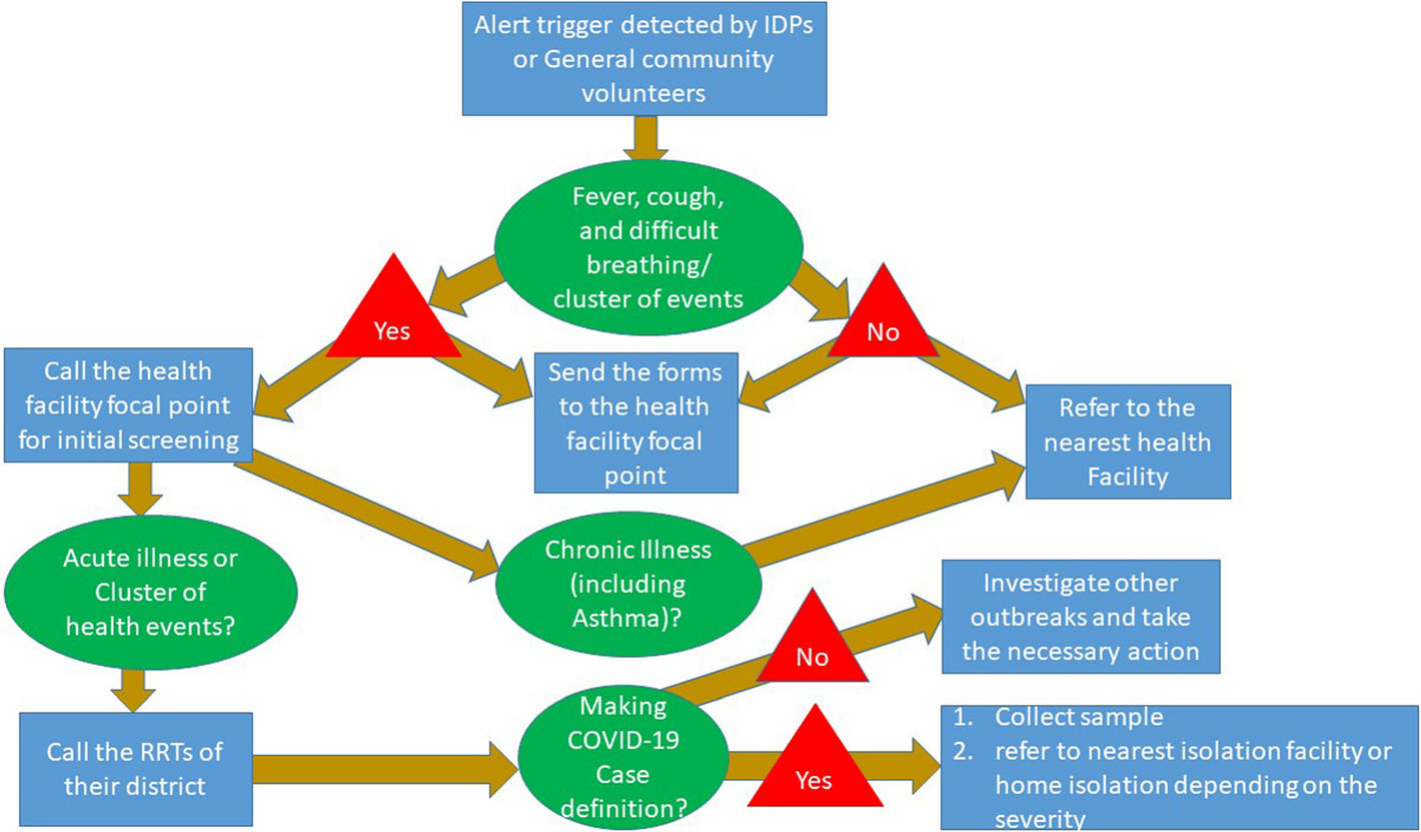
Actions taken to alert triggers identified through community-based surveillance, Yemen, 2020

Immediately after identifying suspected COVID-19 cases and the unusual cluster of diseases, the GCHVs and ICHVs were responsible for contacting the HFFPs through text messages or phone calls and filling in alert trigger forms with detailed information of the cases. The alert trigger form used by the GCHVs and ICHVs included the same information, namely the date of reporting, demographic information of the patient, duration of illness, main signs and symptoms and how the case was detected (see Additional file 1 and Additional file 2). The HFFPs assessed whether the cases required further investigation by RRTs, direct referral to a health facility, or home stay (Fig. 4). The assessment was done through phone calls to the volunteers and patients to obtain additional clinical information and classify the cases according to their severity and public health impact. Potentially severe cases were also visited physically after phone calls. A photo of the alert trigger form was also sent in real time to the HFFP via a WhatsApp message. The HFFP compiled the alert triggers variables in a line list and weekly aggregated form. The weekly aggregated form included the total number of cases, deaths, cases by age group, number of cases by disease, number of cases discovered through door-to-door visits, community meetings and others, and number of cases referred to RRTs, health facilities and advised to stay home (see Additional file 3). The line lists contained all the variables included in the alert trigger form, detailed signs and symptoms, case classification in mild, moderate, or severe, and actions taken for each case. The GCHVs, ICHVs and HFFPs were also requested to produce a weekly narrative report highlighting the main activities implemented during the week and challenges faced in bullet points. Credit for phone calls and internet was provided by WHO to the GCHVs, ICHVs and HFFPs. HFFPs provided feedback about the quality of the reports, accuracy of the alert triggers and findings of the initial assessment of the cases to the GCHVs and ICHVs during weekly coordination meetings.

### Reports submitted

In Hadramout governorate, 18% (14/78) of trained GCHVs submitted at least one alert trigger form to the HFFP (Table 5). Among trained ICHVs in districts selected for CBS implementation, 58% (18/31) submitted at least one alert trigger form (Table 2). The HFFPs from IDP camps submitted 100% of the expected 52 weekly aggregated reports and 10% (*n* = 5) weekly narrative reports. The HFFPs from Al-Mukalla city submitted 65% (*n* = 55) of the expected 84 weekly aggregated reports and 1% (*n* = 1) of weekly narrative reports.

**Table 5:** Community report submission through CBS in Hadramout, Yemen, 2020

Zone	Number of volunteers who sent at least one alert trigger	Percentage of volunteers who sent alert triggers
Buwaish	2	11%
Fowa	4	20%
Al-Deis	6	32%
Alsharej	2	10%
Total	14	18%

*CBS* community-based surveillance

### Alerts detected through CBS and eDEWS

#### Overall alerts

**Fig. 5:**
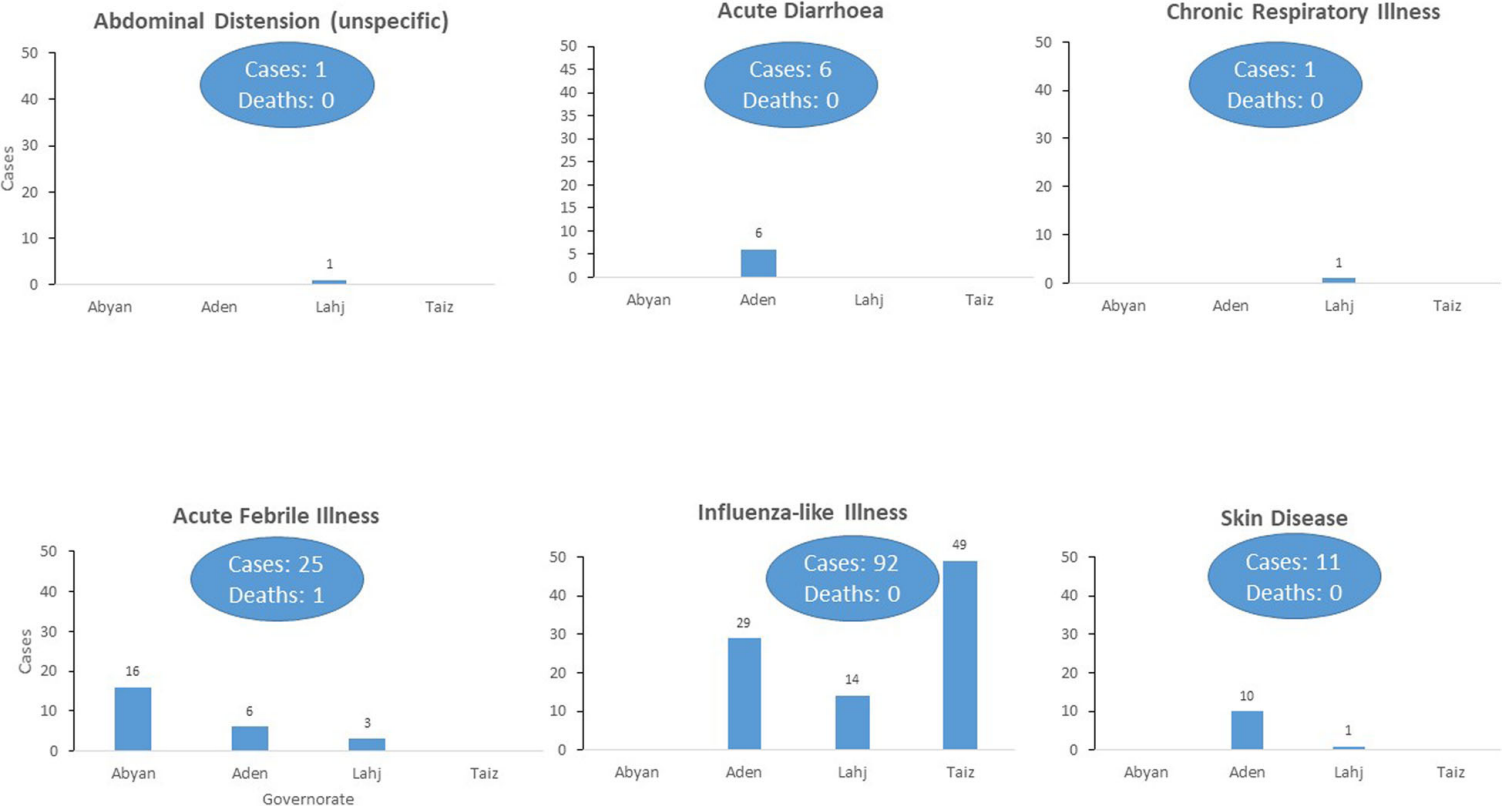
Alerts detected by volunteers from internally displaced people camps, Yemen, 2020

From 10 May 2020 to 24 September 2020, a total of 49 alerts were reported by the GCHVs, all of them influenza-like illnesses. The GCHVs reported no deaths during the same surveillance period. All cases in Hadramout governorate were detected during door-to-door visits and were referred to health facilities and the RRTs were not deployed. From 28 June 2020, when the first alert was reported in IDP camps, to 30 September 2020, 133 alerts were reported by the ICHVs, 69% (*n* = 92) of them being the influenza-like illness and 31% (*n* = 41) being unexplained clusters of diseases or public health events. During the same surveillance period, six main groups of disease alerts, namely influenza-like, chronic respiratory, acute febrile illnesses, acute diarrhoea, skin disease, and abdominal distension, were detected by ICHVs (Fig. 5). The alerts of chronic respiratory illness and abdominal distention had no criteria to be reported by the ICHVs as they did not constitute a cluster of a disease involving two or more people.

#### Influenza-like illness and COVID-19

**Fig. 6:**
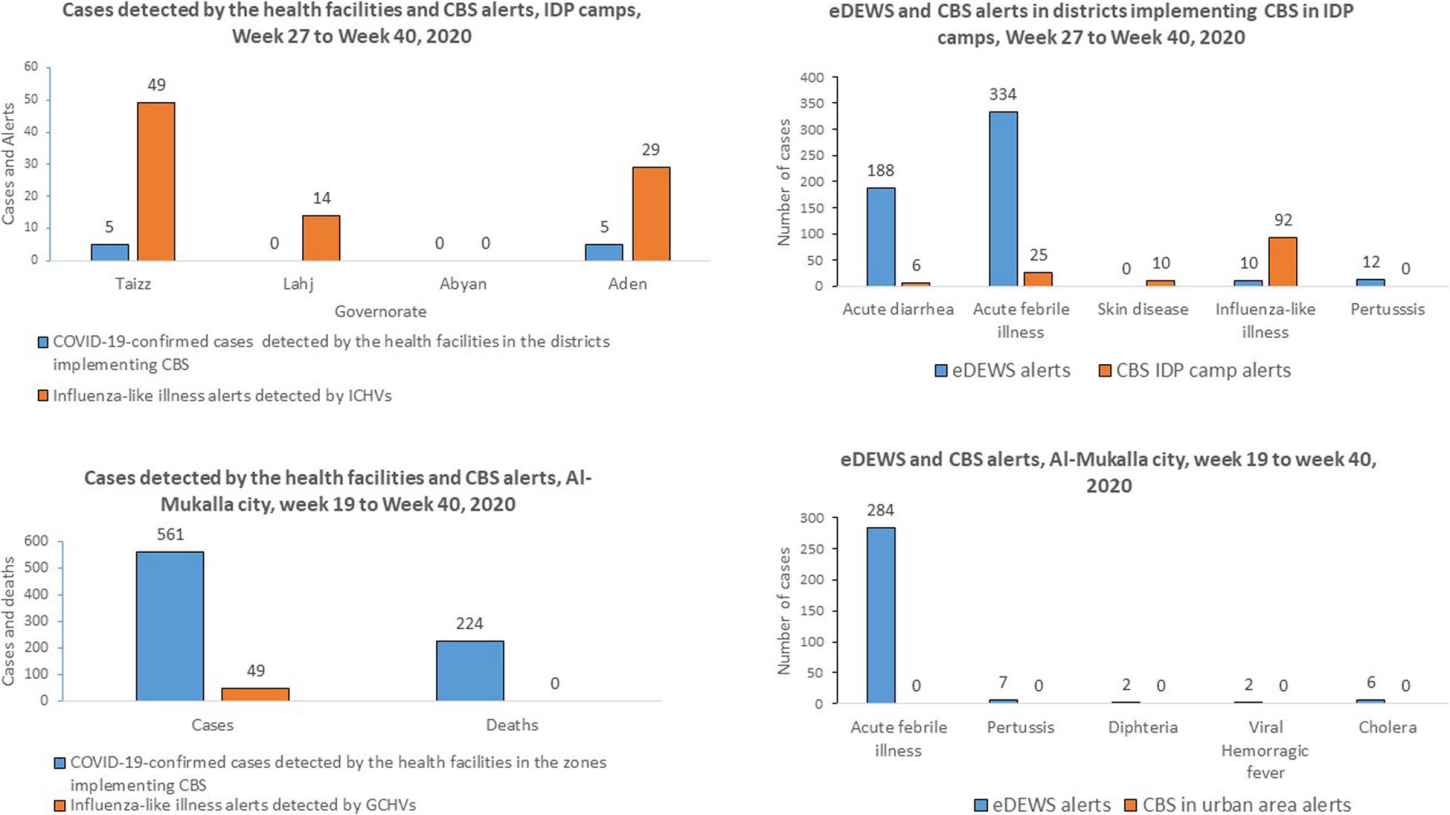
Alerts of influenza-like illness detected through community-based surveillance, Yemen, 2020

From the 49 alerts of influenza-like illness detected by GCHVs from 10 May 2020 to 24 September 2020, information about the severity of the cases, and cases making suspected COVID-19 case definition were not documented in the line list from Al-Mukalla city. The Buwaish district reported 49% of all cases and from all age groups (Table 6). During the same surveillance period, a total of 561 confirmed COVID-19 cases, including 224 deaths (case fatality ratio = 40%) were detected by health facilities in Al-Mukalla city (Fig. 6).

**Table 6:** Cases of influenza-like illness reported through CBS in Hadramout, Yemen, 2020

	Al Share zone	Al-Deis zone	Buwaish zone	Fowah zone	Total
*Sex*					
Female	4	2	6	0	12
Male	10	4	18	5	37
Total	14	6	24	5	49
*Age group, years*					
0–4	2	0	1	0	3
5–9	1	1	4	1	7
10–19	3	2	3	0	8
20–29	1	1	4	0	6
30–39	3	2	3	0	8
40–49	2	0	1	1	4
50–59	2	0	4	2	8
> 60	0	0	4	1	5

*CBS* community-based surveillance

**Fig. 7:**
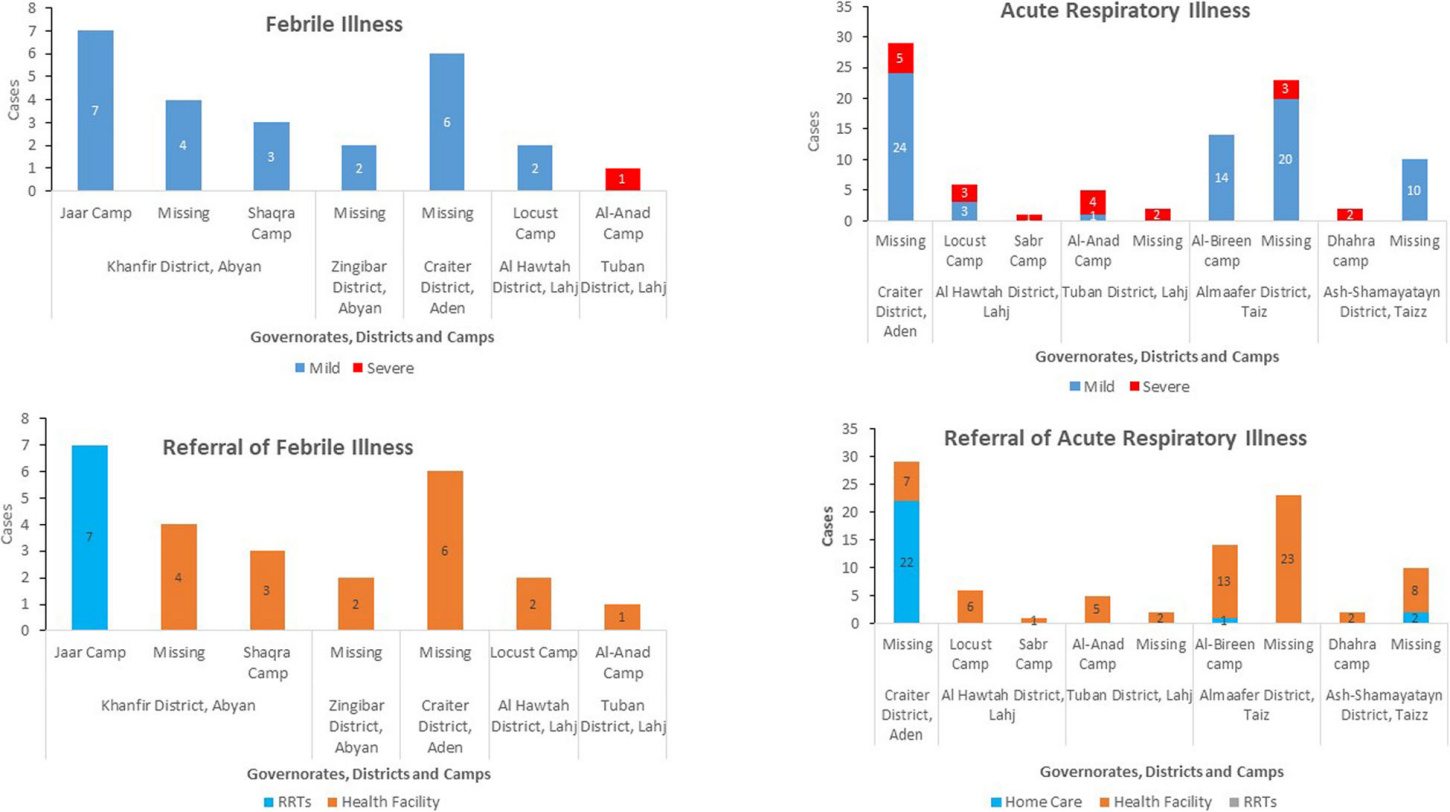
Acute febrile illness and influenza-like illness cases detected though community-based surveillance, 2020

Influenza-like illnesses were the most reported alerts by ICHVs, with 92 cases and 0 deaths reported from 9 IDP camps and 5 districts in Aden, Lahj, and Taiz governorates (Fig. 7). None of them made suspected COVID-19 case definition.

Among the 92 reported cases, 22% (*n* = 20) were severe and 78% (*n* = 72) were mild cases. Among the mild cases, 35% (*n* = 25) were advised to receive care at home. Furthermore, among 92 reported cases, 73% (*n* = 67), including all severe cases, were referred to health facilities. All cases of influenza-like illness were sporadic, and none of them was investigated by RRTs. During the implementation of CBS in IDP camps, 10 COVID-19 cases were detected by the health facilities from three districts implementing CBS: five cases in Craiter district in Aden governorate, two cases in Ash-Shamayatayn, and two cases in Al Ma’afer districts, both in Taiz governorate. None of the cases were referred from IDP camps.

#### Unexplained cluster of diseases

Although the GCHVs did not detect cluster of diseases or public health events besides influenza-like illness, 284 alerts of acute febrile illness were detected through eDEWS in Al-Mukalla city. The city was affected by a dengue fever outbreak. Alerts of suspected measles, pertussis, diphtheria, viral haemorrhagic fever and cholera were also detected through eDEWS during the surveillance period, each of them with less than 10 cases reported.

The ICHVs detected alerts of 25 cases and 1 death owing to acute febrile illness from 7 camps and 5 districts in Abyan, Aden, and Lahj governorates. All the cases were mild, barring a severe case reported in Lahj governorate, which ended in death. Furthermore, all cases were referred to health facilities for treatment. During week 32, one cluster of seven cases of acute febrile illness was detected in a camp in Abyan governorate, affecting children aged < 10 years. The RRTs were called for investigation and response. All the cases were mild, with isolated fever, without other associated symptoms. Four cases were females, and 4 were children aged < 5 years.

A cluster of six cases (no deaths) of acute diarrhoea was detected by ICHVs in a camp in Aden governorate during week 32. All the cases were mild and were reported in four children aged < 5 years and two adults of 28 and 35 years. The RRTs were called for investigation and response.

**Fig. 8:**
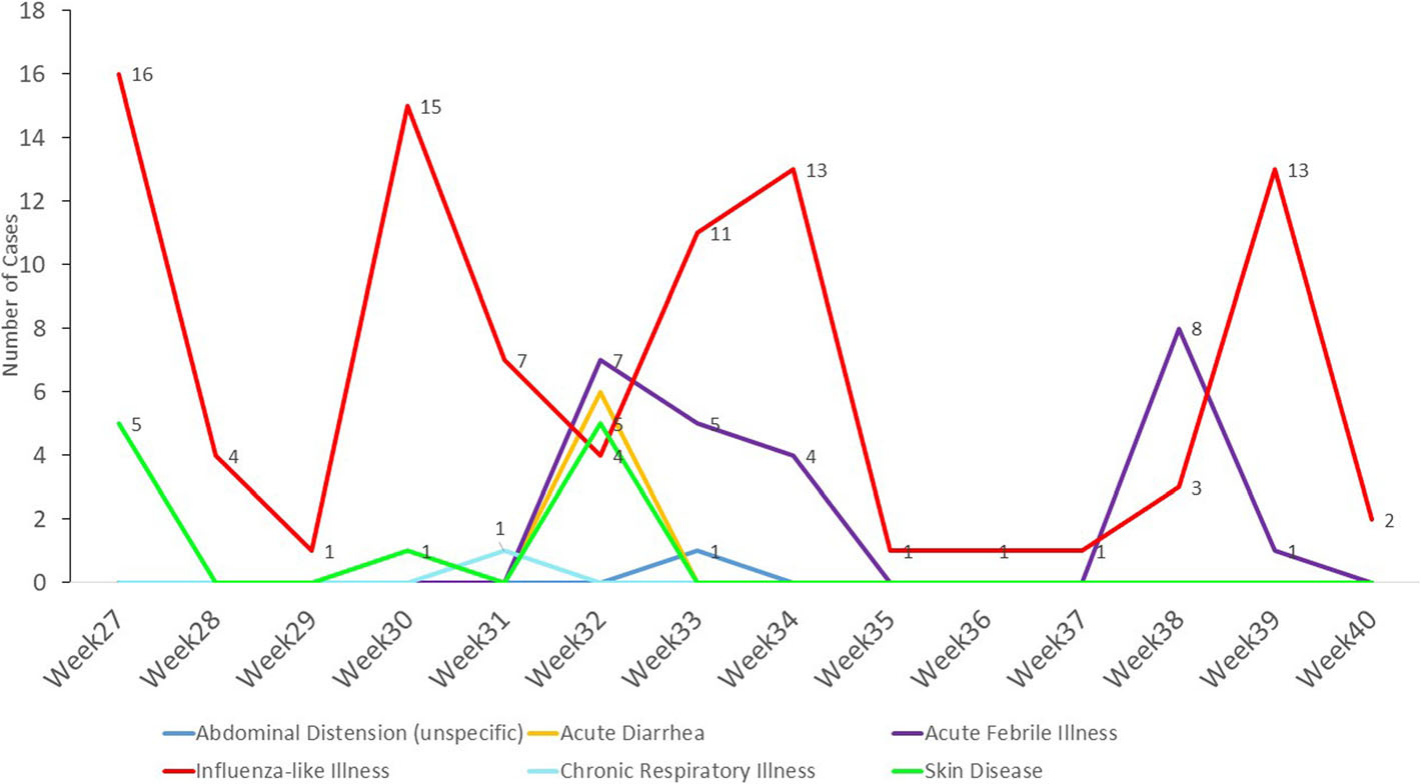
Weekly trends of diseases detected by volunteers from internally displaced people camps, 2020

During weeks 27 and 32, ICHVs also detected two cluster of a mild skin disease in two camps in Aden governorate. The RRTs were called for investigation and response to the two clusters. Each of the clusters affected five people, with 80% of them (*n* = 8) being children aged < 10 years and the remaining being two adults of 18 and 35 years (Fig. 8).

All cases of diseases detected by ICHVs were detected through household visits, except two cases of acute febrile illness and one case of skin disease detected during community meetings. During the same surveillance period, all the districts implementing CBS in IDP camp were affected by dengue fever and cholera outbreaks. Those districts reported a total of 334 alerts of acute febrile illness, 188 alerts of acute watery diarrhoea, 12 alerts of pertussis and 10 alerts of influenza-like illness through eDEWS during the period of CBS implementation. No alert of skin disease alerts were reported through the eDEWS.

#### Public health responses

The cases of influenza-like illness in both IDP camps and urban areas did not trigger the deployment of RRTs and no specific public health actions were taken besides the referral of the cases to the health facilities for further investigations.

In the IDP camps, the cluster of febrile illness, acute diarrhoea and skin diseases were responded by the RRTs. The response to the cluster of acute febrile illness in Abyan governorate included the referral of the cases to the nearest health facility for further investigations, health education to the families on mosquito-borne disease prevention and removal of potential mosquito breeding sites around the houses. For the cluster of acute diarrhoea cases in Aden governorate, the RRTs provided oral rehydration solutions to the households as all the cases were mild, besides the chlorination of the drinking water and health education on prevention of diarrheal diseases. The cases of skin disease detected in Aden governorate were referred to the health facility for further investigation. Additionally, the RRTs provided health to the households on environmental cleaning, personal hygiene, waste disposal, treatment of water and others. No additional cases of the three diseases were reported in the camps after the public health actions were taken.

### Weekly narrative report findings

The main challenges reported in Al-Mukalla city were instances of household refusal to interact with GCHVs owing to limited COVID-19 awareness, and the fear of GCHVs to participate in CBS owing to the lack of PPEs. Furthermore, other challenges reported in Hadramout were limited GCHV knowledge about other diseases and difficulties in performing door-to-door visits owing to frequent lockdowns. HFFPs from IDP camps and ICHVs reported insufficient health facilities near the camps for patient referral, difficulties in inter-camp movements owing to weather conditions, limited community health awareness, insufficient PPEs, and the lack of ambulances to transport cases to health facilities.

## Discussion

The implementation of CBS in Yemen demonstrated to be more useful in IDP camps than in urban settings. In IDP camps, the system could detect suspected outbreaks and guided public health actions for control. However, it failed to detect outbreaks in urban settings. The reporting rate among GCHVs and HFFPs from Al-Mukalla City was also low, suggesting low acceptability of CBS among the staff. However, the acceptability among the communities remained unclear from our findings. At least one alert trigger was submitted by 58% (18/31) of ICHVs, compared to 18% (14/78) of GCHVs in Al-Mukalla city. The low reporting rates of CBS in Al-Mukalla city may have been affected by low willingness of the GCHVs and HFFPs to perform the activities, low mentoring by the supervisors, and high refusal rates by the communities to provide the information. The higher reporting rate of CBS in the IDP camps was likely because a few households were assigned to each ICHV (2–20 per ICHV). IDP camp households likely trusted the ICHVs more because they resided in the same camp and were very well known. The average numbers of households per GCHV in Al-Mukalla city was 180. Those number suggests that when CBS was activated, the GCHVs were probably not well known by the communities and therefore were not received by households owing to a fear of stigma [1]. Moreover, the assignment of a high number of households per GCHV proved to be ineffective in Kenya owing to the limited time for interaction between volunteers and households [23]. During the Ebola virus disease (EVD) outbreak in Sierra Leone, where the population per district is similar to that in Yemen, CBS was successfully implemented because many GCHVs were assigned for each district, with more than 1000 volunteers per district. In Sierra Leone, CBS detected 30% (16/31) of the EVD cases, while 70% of the cases were detected through the regular surveillance system [14]. In addition, GCHVs in Al-Mukalla city probably had the same fear of interacting with people with suspected COVID-19 in activities such as the investigation of community deaths, as the other health workers and RRTs owing to the lack of PPEs, as documented in the beginning of the pandemic in Yemen [1]. Although PPEs were provided by UNICEF and CCCM partners, they were likely insufficient due to global shortage observed during the initial stages of COVID-19 pandemic [24]. Confidentiality issues during the field activities may also have contributed to fear of stigmatization and refusal by the communities in Al-Mukalla city. The GCHVs were oriented to not enter the houses as a precautionary measure. This may have led to fear of the households that neighbours and other people in the streets would hear their conversation with GCHVs. The implementation of CBS in urban areas such as Al-Mukalla city is usually a challenge, requiring better coordination and an extensive education of communities, as demonstrated in Kenya and Yemen during the implementation of integrated community case management program in 2017 [23, 25].

The variety of diseases alerts reported by ICHVs was also higher than those reported by GCHVs from Al-Mukalla city. While 14 GCHVs from Al-Mukalla city only detected alerts of influenza-like illness, 18 ICHVs detected five other syndromes, including suspected outbreaks of a febrile illness, acute diarrhoea, and skin disease, besides influenza-like illness. While GCHVs detected 49 alerts of influenza-like illness, the health facilities in the same area of CBS implementation reported 561 confirmed cases of COVID-19, including 224 deaths (case fatality ratio = 40%). The high mortality of COVID-19 in these areas indicates that the cases reached the health facility late and likely many cases remained in the houses undetected by CBS [1]. Additionally, 90% (*n* = 92) of influenza-like illness alerts detected in the districts implementing CBS in IDP camps were reported by ICHVs, compared to 10% (*n* = 10) of alerts detected through eDEWS during the same surveillance period. The number of people per household in IDP camps is usually higher than in host communities, and people in the IDP camps live in precarious conditions [11]. Therefore, different infectious disease outbreaks are usually anticipated among camp IDPs [26, 27]*.* Furthermore, the districts implementing CBS in IDP camps in Yemen were facing outbreaks of dengue fever and cholera. However, Al-Mukalla city was also facing an outbreak of dengue fever, and 284 alerts of acute febrile illness were reported through eDEWS during the CBS implementation period. Therefore, it would be expected alerts of febrile illness to be reported also by GCHVs. The early detection of suspected outbreaks of acute febrile illness, acute diarrhoea, and skin disease by the ICHVs allowed early deployment of RRTs, implementation of public health actions, and prevented the occurrence of additional cases. Owing to insufficient trainings and inadequate knowledge on other diseases, GCHVs from All-Mukalla city reported only cases of influenza-like illness. ICHVs, despite their insufficient trainings on other diseases, were supervised by medical doctors, and few ICHVs were assigned for each supervisor. In Hadramout governorate, the GCHVs were supervised by non-clinicians who had insufficient clinical knowledge despite their public health background.

To allow for the identification of some challenges prior to CBS implementation, project feasibility and community acceptability should be assessed [6]. However, in Yemen, proper assessments were not performed because the project was implemented as part of an emergency response rather than as routine surveillance. The community and local authorities perceptions regarding CBS, reasons of low reporting of cases to the health facilities, proper calculation of resources and supplies needed, sustainability and other aspects should be considered before the implementation of CBS [25, 28].

The inadequate number of health facilities closer to camps for patient referral, difficult inter-camp movements owing to weather conditions, and lack of ambulances to transport the cases to the health facilities were some of the challenges reported by ICHVs. In both IDP camps and urban settings, no feedback was provided from laboratories and health facilities to CBS focal points. All suspected and confirmed cases were recorded in the generic line lists of routine surveillance, and the cases reported through CBS were not specified. The challenges reported in Yemen were previously documented during the implementation of the integrated community case management in 2017 and are similar to those in other developing countries that attempted CBS implementation [15, 25, 29]. The cost of setting set up a CBS system to support routine surveillance is usually huge, exceeding 1000,000 USD for the purchase of motorbikes, organisation of trainings, purchase of mobile phones and other items, excluding a monthly running costs that may exceed 100,000 USD in a country such as Sierra Leone [14]. Most of the required items, including mobile phones and computers, were not provided in Yemen, which was another probable reason for the inadequate reporting from GCHVs in Al-Mukalla city. However, the impact of CBS in Yemen, especially in IDP camps, was higher when matched with the funds used for its implementation that did not exceed 50,000 USD for 6 months. Limited funds available for CBS continuation is a very commonly reported challenge in other countries [29, 30].

This study had several potential limitations. First, there were no available previous studies documenting CBS in Yemen and the studies from other countries in the region were also few. Therefore, we could not compare our findings with those from other Yemeni and Eastern Mediterranean region studies. Second, some key indicators of CBS such as question refusal rates in the communities, timeliness of data reporting, and quantity of PPEs provided to the volunteers and focal points, were not properly documented. Therefore, we were unable to assess some of the CBS attributes requiring these indicators. Third, the information on the number of households in IDP camps was not accurate because people were constantly arriving and leaving the camps. Therefore, some numbers observed by ICHVs did not reflect those reported in RDT system. Thus, we adjusted the numbers in our report by matching with the numbers provided by the ICHVs. Despite these limitations, our findings will be useful for a further expansion of the project in the future.

## Conclusions and future perspectives

CBS in Yemen demonstrated to be an extremely useful tool to detect outbreaks of respiratory and other diseases in IDP camps, as it led to early detection and implementation of public health actions which prevented the occurrence of further cases. CBS implementation in the early stage of the COVID-19 pandemic, when little was known about the disease, did not yield expected results in general communities in urban areas. The system failed to detect suspected COVID-19 cases and other diseases in Al-Mukalla city despite the ongoing outbreaks in the same location reported through eDEWS. The ability of the staff, including the detection of diseases in the communities was better among volunteers supervised by clinicians than among those supervised with staff without a clinical background. The acceptability of CBS among the staff was higher in IDP camps compared to the urban settings. The acceptability among the communities was unclear from our findings. In Yemen, as in other countries, feasibility and acceptability studies should be conducted few months before CBS expansion in urban communities. Due to the ongoing complex emergency and COVID-19 pandemic, CBS should be expanded in IDP camps in Yemen, through creation of suspected COVID-19 and other diseases outbreaks reporting sites. We also recommend the volunteers to be supervised by staff with clinical background; development of country specific CBS guidelines with community case definitions for other diseases with a risk for outbreak in Yemen; and the strengthening of the link between CBS and routine surveillance systems through feedbacks to HFFPs and community volunteers on the outcome of patients and laboratory results.

## Supplementary Information

**Additional file 1.** Alert trigger form in English translation.

**Additional file 2.** Alert trigger form in original Arabic version.

**Additional file 3.** Weekly aggregated form.

## Data Availability

All data generated or analysed during this study are included in this published article.

## Abbreviations

C4D: Communication for development
CBS: Community-based surveillance
CCCM: Camp Coordination and Camp Management
COVID-19: Coronavirus disease - 19
eDEWS: Electronic disease Early Warning System
EVD: Ebola virus disease
GCHV: General Community health volunteers
GHO: Governorate Health Office
HFFP: Health facility focal points
ICHV: IDP community health volunteer
IDP: Internally displaced people
IOM: International Organization for Migration
MOPHP: Ministry of Public Health and Population
PPE: Personal protective equipment
RDT: Rapid displacement tracking
RRT: Rapid Response Team
UNICEF: United Nations Children’s Fund
WHO: World Health Organization
