# Supplementary material for: Community-based surveillance in internally displaced people’s camps and urban settings during a complex emergency in Yemen in 2020
Source: Confl Health. 2021 Jul 5;15:54. doi: 10.1186/s13031-021-00394-1 (PMC8256204; doi:10.1186/s13031-021-00394-1)
Supplement: Supplementary file 1 — Additional file 1. Alert trigger form in English translation. [file 13031_2021_394_MOESM1_ESM.pptx]

## Slide 1
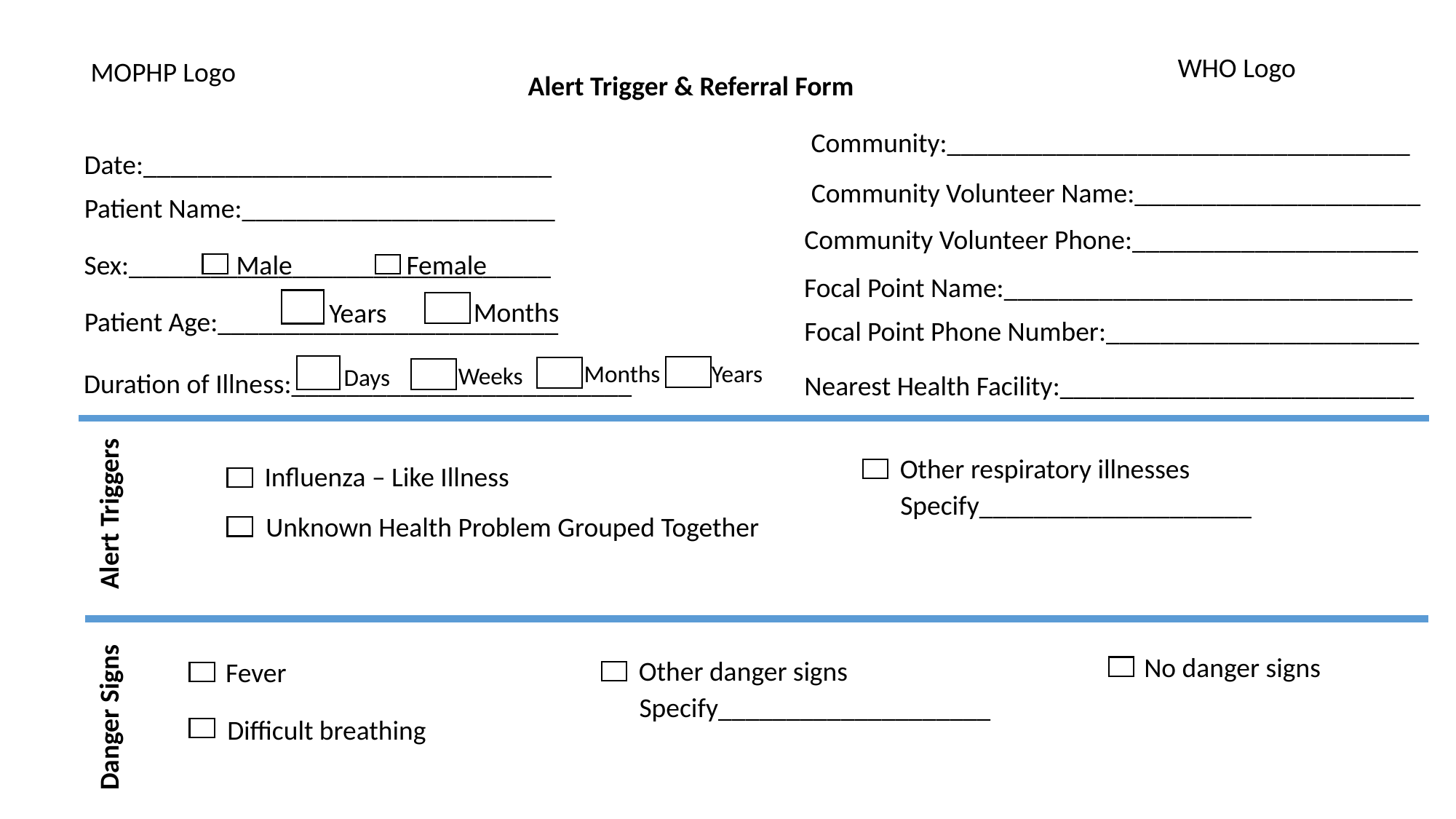

WHO Logo
MOPHP Logo
Alert Trigger & Referral Form
Community:__________________________________
Date:______________________________
Community Volunteer Name:_____________________
Patient Name:_______________________
Community Volunteer Phone:_____________________
Sex:_______________________________
Male
Female
Focal Point Name:______________________________
Months
Years
Patient Age:_________________________
Focal Point Phone Number:_______________________
Months
Years
Weeks
Days
Duration of Illness:_________________________
Nearest Health Facility:__________________________
Other respiratory illnesses
Influenza – Like Illness
Specify____________________
Alert Triggers
Unknown Health Problem Grouped Together
No danger signs
Other danger signs
Fever
Specify____________________
Danger Signs
Difficult breathing

## Slide 2
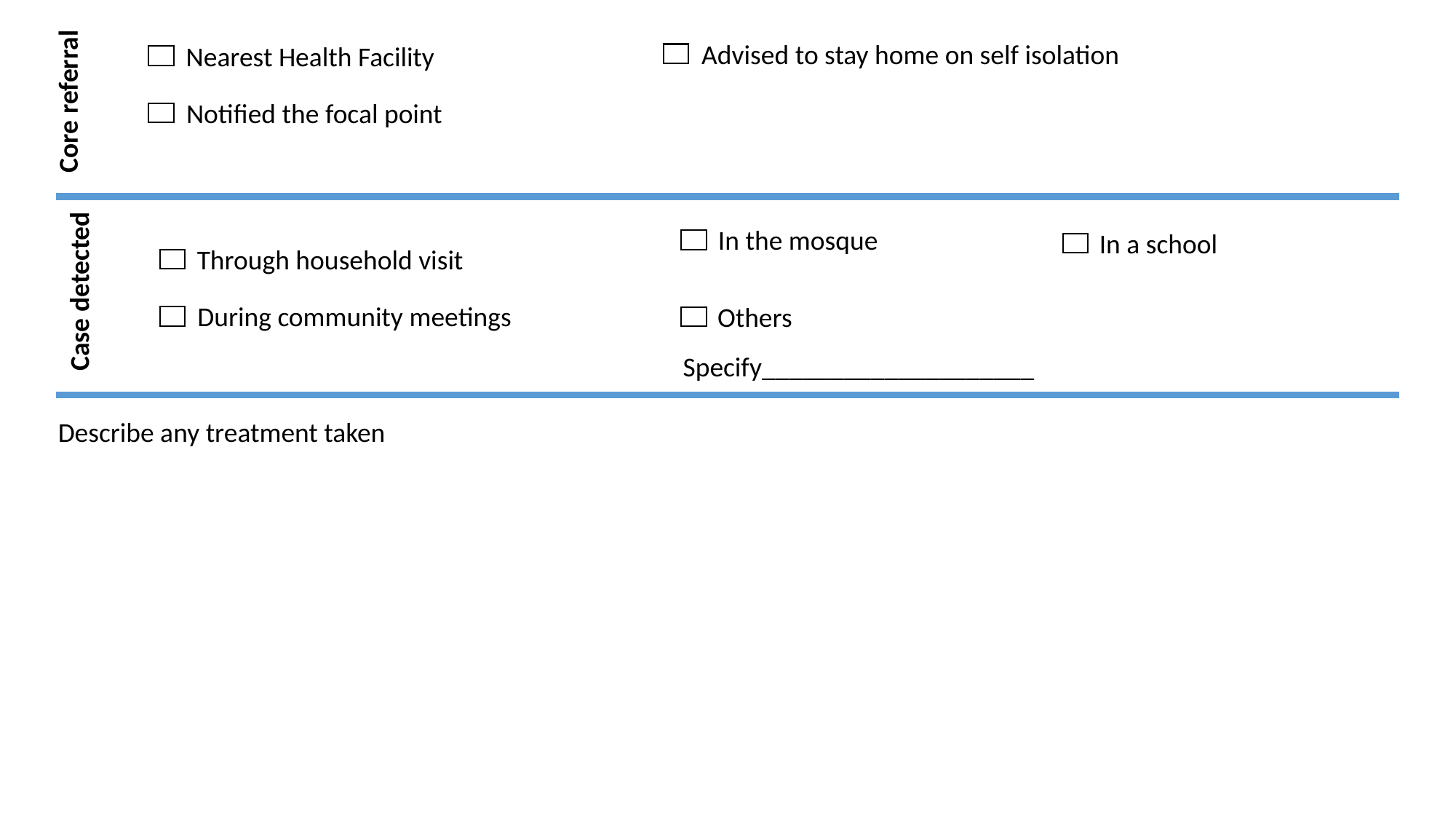

Advised to stay home on self isolation
Nearest Health Facility
Core referral
Notified the focal point
In the mosque
In a school
Through household visit
Case detected
During community meetings
Others
Specify____________________
Describe any treatment taken
