# Supplementary material for: Community-based surveillance in internally displaced people’s camps and urban settings during a complex emergency in Yemen in 2020
Source: Confl Health. 2021 Jul 5;15:54. doi: 10.1186/s13031-021-00394-1 (PMC8256204; doi:10.1186/s13031-021-00394-1)
Supplement: Supplementary file 2 — Additional file 2. Alert trigger form in original Arabic version. [file 13031_2021_394_MOESM2_ESM.pdf]

استمارة جمع البيانات على مستوى المتطوع المجتمعي

التاريخ: ....

اسم المريض: ....

الجنس: ذكر ☐ انثى ☐

عمر المريض:

فترة ظهور الاعراض: ☐ايام ☐ اسابيع شهور☐

سنوات ☐

اسم الموقع: ....

اسم متطوع المجتمع: ....

رقم متطوع المجتمع: ....

اسم المشرف: ....

رقم المشرف: ....

اسم اقرب مرفق صحي: ....

هل يوجد اشتباه انفلونزا: نعم ☐ لا ☐

هل يوجد مرض غير اعتيادي: نعم ☐ لا ☐

اذا كانت الاجابة نعم, اكتب ماهي الاعراض:

هل يعاني الشخص حمى: نعم لا ☐

هل يعاني الشخص من صعوبة في التنفس: نعم ☐ لا ☐

هل يعاني الشخص من سعال: نعم ☐ لا ☐

هل يعاني الشخص من اعراض اخرى: نعم ☐ لا☐

اذا نعم؟ عددها: ....

هل تم اكتشاف الحالة من خلال البحث بالزيارات المنزلية: نعم ☐ لا ☐

هل تم اكتشاف الحالة اثناء الاجتماعات: نعم ☐ لا ☐

هل تم اكتشاف الحالة اثناء الصلاة في المسجد: نعم ☐ لا ☐

هل تم اكتشاف الحالة في المدرسة: نعم ☐ لا ☐

اخرى: ....

هل تمت الاحالة الى المرفق الصحي: نعم ☐ لا ☐

هل تم تبليغ المشرف : نعم ☐ لا ☐
